# Comment on “Dexamethasone Inhibits Spheroid Formation of Thyroid Cancer Cells Exposed to Simulated Microgravity”

**DOI:** 10.3390/cells9071738

**Published:** 2020-07-21

**Authors:** Joseph J. Bevelacqua, James Welsh, S.M.J. Mortazavi

**Affiliations:** 1 Bevelacqua Resources, Richland, WA 99352, USA; bevelresou@aol.com; 2 Department of Radiation Oncology, Edward Hines Jr VA Hospital, Hines, IL 60141, USA; James.Welsh@va.gov; 3 Diagnostic Imaging Department, Fox Chase Cancer Center, Philadelphia, PA 19111, USA

**Keywords:** dexamethasone, thyroid cancer, microgravity, space environment

This letter addresses our concerns about a paper by Melnik et al. entitled “Dexamethasone Inhibits Spheroid Formation of Thyroid Cancer Cells Exposed to Simulated Microgravity” that was published in *Cells* [1]. Melnik et al. have used synthetic glucocorticoid dexamethasone (DEX) to suppress the formation of spheroids in a culture of follicular thyroid cancer (FTC)-133 cells after exposure to simulated microgravity. Despite strengths, this paper suffers from a few shortcomings. The first shortcoming of this paper comes from its simulation protocol. The authors state that they used a random positioning machine (RPM) for simulating microgravity: “The used desktop-RPM (Dutch Space, Leiden, The Netherlands) was located in an incubator with 37 °C/5% CO_2_ and operated in real random mode, with a constant angular velocity of 60°/s”. RPMs that represent the more sophisticated development of the single-axis clinostats are laboratory instruments that provide a continuous random change in orientation relative to the gravity vector of an accommodated (biological) experiment. Given this consideration, RPMs usually consist of two independently rotating frames (the second frame is positioned inside the first frame) to give a very complex net of orientation change to a biological sample. RPMs cannot really simulate microgravity, and as well as this the cells experience strong shearing forces and microscopic friction that can significantly affect the findings of this study. It is worth noting that the convection and shear stresses that occur inside a cell culture flask during RPM experiments are addressed in previous studies: “The RPM rotation introduces fluid motion in the culture flask, leading to shear forces and enhanced convection” [2]. Along the flask walls, shear stresses can reach up to a few 100 mPa depending on the rotational velocity [3]. Therefore, the fluid dynamic in the culture flasks and its potential effects on cells inside culture flasks turning on an operating RPM should be fully studied.

Moreover, in an actual space environment, the interactions of the key stressors such as radiation and microgravity play a basic role in biological effects, but a single stressor like microgravity does not. The issue of the possible interactions of the space stressors, such as radiation and microgravity, dates back to 1999 [4]. More advanced approaches occurred in the period 2003–2004 [5,6], and this topic still receives great attention [7,8].

In addition, readers of the paper authored by Melnik et al. should be aware of the following points:

1. It is not possible to simulate microgravity on earth. The 9.8 m/s^2^ gravitational field directed towards the center of the planet cannot be eliminated and contributed to the experiment. Given this consideration, microgravity simulation studies conducted using RPMs suffer from two major limitations:

*I.*  Physical Limitations:

Gravity is an interaction that is based on the distance between masses. For Earth, the gravitational interaction and its gradient can be approximated by a direction with respect to the center of the Earth. Since that distance is large, there is not much of a surface gradient. When that distance is shortened, the gradient becomes more severe because the acceleration varies with the distance to the various parts of a body. Acceleration is a vector quantity. This effect increases as the body size increases. It is negligible for a point source, but not so for an extended body.

Given this consideration, while on earth, a preferred direction is defined by the planet’s gravitational field of 9.8 m/s^2^, whereas in space there is no preferred direction. Thus, simply we cannot do an experiment on Earth to negate this field for an extended body.

A random positioning machine (RPM) can, in principle, establish an oppositely directed acceleration for an instant at a particular body location (or a particular point of a biological sample, cell culture, etc.), but the rest of the body/sample is subjected to the Earth’s gravity, or a portion of it. Therefore, parts of the body/sample still experience an acceleration. For an extended body, the RPM does not uniformly negate gravity, and various body parts receive various accelerations. This is not representative of a microgravity environment. Moreover, the duration of the RPM usage is much shorter than a space mission, particularly an extended International Space Station (ISS) or Mars mission.

*II.*  Biological Limitations:

The RPM device subjects the body to stresses that are notencountered in microgravity. These stresses have an effect on biological processes that differ from a microgravity environment. In particular, cells will experience strong shearing forces and microscopic friction. 

In summary, the classic error is assuming that if a vector sum of acceleration is zero, it can be equated to zero gravity. This may be true for a point particle, but not an extended system. The physics does not yield zero, and the biology certainly differs. In other words, the Earth’s gravitational force is balanced by a centripetal force, mv^2^/r, where r is the distance to the center of the device. This distance variation creates the gradient. It does not lead to a significant gradient for the Earth, but it does for the smaller RPM machines. This gradient is important because the microgravity is so small. Therefore, any gradient is significant compared to microgravity.

2. The preferred direction produced by the field is not equivalent to a microgravity non-directional field that is encountered in space. 

3. Individual cells do not necessarily provide specific data regarding cancer in an organism or specific organ. The immune system, DNA repair mechanisms, and whole-body response are not equivalent to individual cellular response data. This is part of the linear non-threshold fallacy that uses single cell data to derive the biological response in an organism. Cell data are important, but also must be properly characterized by including the mitigating measures associated with the collective human biological repair mechanisms.

Readers of [1] should be aware of the limitations of performing Earth-based experiments to simulate microgravity. The experimental simulations and the Earth’s inherent gravitational field and associated forces are not equivalent to the space microgravity environment. In addition, the space radiation environment is not equivalent to the Earth-based radiation environment. Evaluating the effects of microgravity in space must also address the space radiation environment to provide a comprehensive evaluation of the presumed biological effects under investigation.
